# Editorial: Moving beyond the molecular mechanisms of malignant pleural mesothelioma: Cues for novel biomarkers and drug targets

**DOI:** 10.3389/fonc.2023.1163144

**Published:** 2023-03-06

**Authors:** Ilaria Cavallari, Ferdinando Cerciello, Elisa Giovannetti, Loredana Urso

**Affiliations:** ^1^ Immunology and Molecular Oncology Unit, Istituto Oncologico Veneto – Istituto di Ricovero e Cura a Carattere Scientifico (IRCCS), Padua, Italy; ^2^ Department of Medical Oncology, Inselspital, Bern University Hospital, University of Bern, Bern, Switzerland; ^3^ Laboratory Medical Oncology, Department Medical Oncology, Cancer Center Amsterdam, Amsterdam Universitair Medische Centra (UMC), Vrije Universiteit, Amsterdam, Netherlands; ^4^ Cancer Pharmacology Lab, Fondazione Pisana per la Scienza, Pisa, Italy; ^5^ Department of Surgery, Oncology and Gastroenterology, University of Padua, Padua, Italy

**Keywords:** MPM, biomarkers, target therapies, omics data analysis, data-repository

Malignant pleural mesothelioma (MPM) is an important challenge for our society and world health even though it is a rare tumor. Its major cause, asbestos, has been abundantly utilized in the last century, and it is now widely present in our daily environment, for example, in constructions. Although most of the high-income countries have banned asbestos, larger parts of the world’s population are still exposed in an uncontrolled way to the mineral, and worldwide the incidence of MPM is rising (1, 2). In parallel, progresses on the therapies available to treat MPM are advancing only at a low pace, and cure remains elusive (3). For almost two decades, platinum and pemetrexed (PMX) remained the treatment cornerstone and it is only recently that immunotherapies are offering an alternative (4, 5). The biology of MPM is complex, and its clinical presentation is challenging, but these are not the sole reasons for such slow progress. MPM is a rare cancer, making it less attractive for experimenting with novel technologies or innovative approaches. For example, dedicated data repositories or bioinformatic strategies, in particular in the field of novel omics sciences, are rare in MPM, limiting the access to important resources for biological and translational studies or novel biomarkers. We recognize that in the era of modern research resulting in a high amount of accessible data, bioinformatics strategies and dedicated data repositories are becoming crucial for analyzing and interpreting the available data in MPM. Within the topic, we therefore intended to provide a space for research works in MPM that may have taken advantage of modern large dataset approaches to improve treatment or biomarker strategies. We highlighted the efforts ongoing within the community to make such approaches possible. We further identified examples of real-world questions in MPM that may profit of the application of such approaches.

In their work, Sato et al. make advantage of RNA sequencing and metabolic approaches to explore resistant mechanisms to PMX. In previous works, low expression levels of thymidylate synthase (TYMS) were significantly associated with disease control, improved progression-free survival, and overall survival in MPM patients treated with pemetrexed-based regimens (6). Keeping with these findings, the present study demonstrates that overexpression of TYMS confers resistance to PMX treatment in MPM cell lines (Sato et al.). Notably, the authors demonstrate by metabolomics analysis that cells resistant to PMX show a reduction in deoxyuridine monophosphate (dUMP) and an increase in deoxythymidine monophosphate (dTMP), suggesting that measurement of dTMP may be an additional biomarker for monitoring PMX response. Thus, prospective trials for the validation of the prognostic/predictive role of both TYMS and dTMP in MPM patients treated with pemetrexed-based regimens are warranted. Among the articles included in this Research Topic, Huo et al. present an excellent example of the usefulness of the few omics data repositories available for translational research in MPM. Using a public dataset available from TCGA, they demonstrate that MPM tumors overexpress TWF1 (Twinfilin Actin Binding Protein 1) and that higher expression is related to a worse prognosis. Other than offering a new biomarker for the prognostic stratification of MPM patients, this knowledge paves the way for investigating the role of TWF1 in MPM biology and its potential as a therapeutic target. To further address the importance of omics data resources for MPM, Martens et al. present the effort of a larger group of MPM and bioinformatic experts who worked together to establish the first version of a molecular pathway model of MPM. The work offers to the community the first pathway knowledge base entirely dedicated to MPM and within an interactive platform that permits and encourages the direct active contribution of each scientist in the field. It is an open-source resource for systems biology-oriented information that may support translational research in MPM. The works of Usada and colleagues (Usada et al.) and Idkedek et al. report examples of real-case scenarios in MPM where large dataset approaches, if routinely in place for MPM, may be beneficial for concrete clinical questions like how to discriminate MPM from confounding diseases or how to recognize individuals hereditary at risk for MPM and how to monitor them for the disease.

In conclusion, this Research Topic aims at giving an overview of MPM, with a special focus on new potential biomarkers and innovative multidisciplinary research strategies against vulnerabilities emerging from omics studies.

## Author contributions

LU invited coeditors. IC, FC, EG, and LU planned the Research Topic, invited the authors, edited papers, and finally wrote the editorial. All authors approved the submitted version.

## References

[1] Alpert N van Gerwen M Taioli E. Epidemiology of mesothelioma in the 21(st) century in Europe and the united states, 40 years after restricted/banned asbestos use. Transl Lung Cancer Res (2020) 9(Suppl 1):S28–38. doi: 10.21037/tlcr.2019.11.11 PMC7082259 32206568

[2] Han Y Zhang T Chen H Yang X. Global magnitude and temporal trend of mesothelioma burden along with the contribution of occupational asbestos exposure in 204 countries and territories from 1990 to 2019: Results from the global burden of disease study 2019. Crit Rev Oncol Hematol (2022) 179:103821. doi: 10.1016/j.critrevonc.2022.103821 36165817

[3] Tsao AS Pass HI Rimner A Mansfield AS. New era for malignant pleural mesothelioma: Updates on therapeutic options. J Clin Oncol (2022) 40 (6):681–92. doi: 10.1200/jco.21.01567 PMC8853621 34985934

[4] Vogelzang NJ Rusthoven JJ Symanowski J Denham C Kaukel E Ruffie P. Phase III study of pemetrexed in combination with cisplatin versus cisplatin alone in patients with malignant pleural mesothelioma. J Clin Oncol (2003) 21 (14):2636–44. doi: 10.1200/JCO.2003.11.136 12860938

[5] Baas P Scherpereel A Nowak AK Fujimoto N Peters S Tsao AS. First-line nivolumab plus ipilimumab in unresectable malignant pleural mesothelioma (CheckMate 743): a multicentre, randomised, open-label, phase 3 trial. Lancet (2021) 397 (10272):375–86. doi: 10.1016/s0140-6736(20)32714-8 33485464

[6] Zucali PA Giovannetti E Destro A Mencoboni M Ceresoli GL Gianoncelli L. Thymidylate synthase and excision repair cross-complementing group-1 as predictors of responsiveness in mesothelioma patients treated with pemetrexed/carboplatin. Clin Cancer Res (2011) 17 (8):2581–90. doi: 10.1158/1078-0432.ccr-10-2873 21262916

